# Are Patients Aware of Clozapine Side Effects?

**DOI:** 10.1192/bjo.2024.372

**Published:** 2024-08-01

**Authors:** Charlotte Golding, Neeti Sud

**Affiliations:** Cumbria, Northumberland, Tyne and Wear NHS Foundation Trust, Newcastle upon Tyne, United Kingdom

## Abstract

**Aims:**

Patients should have a comprehensive understanding of the side effects, and monitoring requirements of the medications prescribed to them. Making the patient aware of serious side effects is important for patient safety and informed consent. Patients should know when and how to seek help for side effects. Health literacy also increases patient autonomy and shared decision making.

As an inpatient, a psychiatric patient’s medications are closely monitored, and there is frequent contact with healthcare professionals who can identify any health needs. Within our trust, there is a side effect checklist to be completed by community staff each time a community patient has clozapine monitoring. However, in our clinical practice, we have observed that some patients have needed prompts regarding need for re-titration if dose missed for 48 hours.

We aimed to assess medication safety information awareness in a small sample of patients open to forensic community team who are prescribed clozapine.

**Methods:**

A 26-point questionnaire was used to assess the participant's depth of knowledge of clozapine. A combination of 3 open and 22 closed questions were used. Patients were scored for their answers to the closed questions, using a predetermined marking scheme, being awarded 1 point per appropriate answer. We set the standard as maximum score of 22.

All participants (n = 7) were male and had been prescribed clozapine for at least one year.

**Results:**

All participants were able to accurately state why they were prescribed clozapine. The mean score was 16. Zero participants scored 22. Lowest score was 14. One participant omitted two questions (Do you know what to do if you take more clozapine tablets than you are supposed to? Do you know what to do if you forget to take clozapine?). He stated that he was very careful regarding his medication and therefore, will not forget or miss any doses.

71% of patients were unsure what they should do if they were to accidentally take more tablets than prescribed.

Five out of seven participants were able to cite at least one side effect of clozapine without prompting.

Two patients were not able to spontaneously recall the monthly blood test requirement.

**Conclusion:**

There was a range of knowledge deficits about clozapine in our sample. After including reminders of safety information about clozapine at quarterly care coordination reviews, we plan to re-assess in a year's time.

